# Commentary: Yin and Yang of MAITs in HCC: balancing type 1 vs. type 17 signaling

**DOI:** 10.1038/s41423-026-01416-9

**Published:** 2026-04-24

**Authors:** Sarah Beyer, Benjamin Ruf

**Affiliations:** 1 Department of Internal Medicine I, University Hospital Tübingen, Eberhard Karls University of Tübingen, Tübingen, Germany; 2 M3 Research Center for Malignome, Metabolome and Microbiome, Faculty of Medicine, University Tübingen, Tübingen, Germany; 3 Cluster of Excellence iFIT (EXC 2180) “Image-Guided and Functionally Instructed Tumor Therapies”, University of Tübingen, Tübingen, Germany

**Keywords:** Tumour immunology, Immune evasion

Mucosal-associated invariant T (MAIT) cells are unconventional or innate-like T cells (ILTCs) that are enriched in the human liver, where they can represent up to 40–50% of all intrahepatic T cells [1]. However, their contribution to tumor immunity in hepatocellular carcinoma (HCC) remains controversial. Both antitumor and protumor effects have been documented, but the cellular heterogeneity underlying this dichotomy is poorly understood [2]. In a new study by Fu et al. [3], published in this issue of *Cellular & Molecular Immunology*, a piece of this puzzle was resolved by demonstrating that MAIT cells possess phenotypic plasticity, giving rise to a distinct T_H_17-polarized CD4^+^ MAIT subset that represents the dominant source of interleukin-17A (IL-17A)-producing lymphocytes in human HCC tumors. The authors demonstrate that IL-17A directly promotes HCC tumor cell proliferation in vitro and lipid storage through a PPARα-dependent metabolic axis. In addition, this study revealed bidirectional metabolic crosstalk in which tumor-derived kynurenine (Kyn) activated the aryl hydrocarbon receptor (AHR) on CD4^+^ MAITs, enhancing glycolysis and increasing IL-17A production. These findings suggest that MAIT cells act not only as innate sentinels but also as plastic effectors that can also contribute to the tumor-permissive tumor microenvironment (TME) to support HCC progression (Fig. 1).

**Fig. 1:**
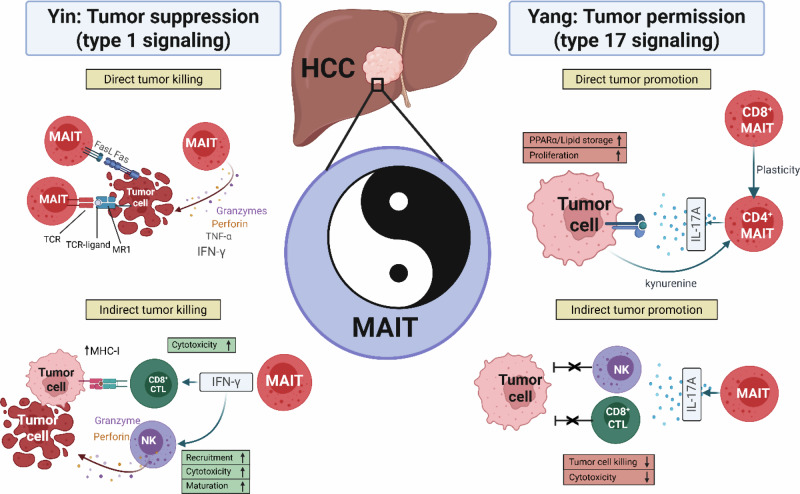
Yin (tumor suppression) and Yang (tumor promotion) of MAITs in HCC: type 1 vs. type 17 signaling: MAIT cells undergo TCR-dependent and TCR-independent activation within the HCC TME, resulting in context-dependent effector functions. On the one hand, MAIT cells can directly lyse tumor cells through granzymes and perforin, TNF-α, IFN-γ or FasL-dependent mechanisms. IFN-γ-producing MAIT cells also promote indirect tumor suppression through other effector cells, such as CD8^+^ and NK cells. On the other hand, in a tumor-permissive TME, MAIT cells can acquire tumor-promoting effector functions. Fu et al. demonstrated that through bidirectional metabolic cross talk, CD4^+^IL-17A-producing MAITs can directly promote tumor cell proliferation. Indirect tumor promotion by IL-17A-producing MAITs in mice converges in terms of CD8^+^ and NK cell suppression

Human MAIT cells recognize microbial riboflavin metabolites presented by the monomorphic MHC class I-related molecule MR1 and, upon activation, rapidly deploy cytokines, including IFN-γ, TNF-α, and IL-17A, as well as cytolytic effector molecules such as granzymes and perforin. The transcriptional program of human MAIT cells is governed by the master transcription factor PLZF as well as T-bet and RORγt, which results in mixed type 1 and type 17 functional phenotypes [4] that are shaped by the tissue environment [5]. In mice, mutually exclusive type 1 and type 17 MAIT cell lineages develop prior to thymic egress (termed MAIT1 vs. MAIT17) [6].

As mainly tissue-residing lymphocytes, their abundance in the liver positions them as potential early sensors of oncogenic transformation, but their functional trajectory within the TME is highly context dependent. While MAIT cells can contribute to antitumor immunity, some clinical data have indicated that activated and exhausted MAIT cells are associated with poor prognosis in patients with HCC, suggesting that these cells potentially contribute to a tumor-permissive microenvironment [7]. In the HCC TME, MAITs are shaped by cellular interactions with parenchymal and immune cells, some of which contribute to a dysfunctional MAIT cell phenotype, such as CSF1R^+^PD-L1^+^ tumor-associated macrophages (TAMs), highlighting PD-1/PD-L1-dependent dysfunction [8]. The contribution of MAIT17 cells to an immunosuppressive TME has previously been demonstrated in mice, where MAIT cells promote tumor initiation, tumor growth, and experimental lung metastasis in murine models, partly through the suppression of NK cell function mediated by IL-17A [9]. In addition to IL-17A-producing MAIT cells, FOXP3^+^CXCR3^+^ MAIT cells in HCC patients were identified as regulatory MAIT cells (and thus termed MAITregs) that show immunosuppressive potential similar to that of conventional T_regs_ [10].

Conversely, MAIT cells have been associated with better clinical outcomes in HCC cohorts and murine models [8]. When activated in a TCR-dependent and/or TCR-independent fashion, MAIT cells can directly lyse tumor cells in vitro through IFN-γ-, TNF-α- and FasL-dependent mechanisms [11]. From a therapeutic perspective, MAIT cells can be targeted by agonistic T-cell receptor (TCR) ligands derived from the metabolism of riboflavin, such as 5-OP-RU. Pharmacological costimulation with the MAIT agonist 5-OP-RU and (in some studies) the TLR9 agonist CpG unleashes potent antitumor immunity across multiple murine models, switching MAIT cells from a T_H_17-like to a T_H_1/NK-like phenotype [12, 13]. Taken together, these studies revealed that MAIT cell function is not fixed but is exquisitely tuned by activation signals, the TME, and checkpoint pathways; however, the precise mechanisms through which the TME remodels MAIT cell identity remain unclear. Contradictory findings linking MAIT cells and tumor outcome highlight our limited knowledge about the function of MAITs in the context of liver cancer and the previously limited granularity of analyses of the MAIT cell phenotype.

## A Th17-polarized CD4^+^ MAIT subset is differentiated from CD8^+^ MAITs

Fu et al. established that in human HCC tumors, IL-17A-producing T cells are predominantly MAITs, specifically the CD4^+^ MAIT subset. This CD4^+^ IL-17A-expressing MAIT subset is enriched in HCC tumors relative to that in paired nontumor adjacent liver tissues. While the accumulation of CD4^+^ MAIT cells in HCC tumor lesions has previously been described [8], the functional role of the CD4^+^, CD8^+^ and double-negative (DN) MAIT cell subsets is unclear. The authors now demonstrate the phenotypic plasticity of human MAIT cells by using sorted MAIT cell subsets and multiple activation protocols: TCR-dependent (anti-CD3/CD28, MR1-5-OP-RU tetramer) as well as TCR-independent (IL-12 plus IL-18) stimulation resulted in CD4^+^ MAITs that differentiated from sorted CD8^+^ MAITs via a CD4^+^CD8^+^ double-positive intermediate population. Trajectory analysis and shared TCR clonotypes between CD4^+^ and CD8^+^ MAITs in tumor tissue provide evidence for the shared ontogeny of these lineages. As the supernatant from LM3 hepatoma cells accelerated this CD8^+^ → CD4^+^ MAIT conversion, tumor-secreted factors were shown to actively promote this phenotypic plasticity.

## Pro-tumor effector mechanism: IL-17A, PPARα, and lipid metabolism

IL-17A-producing MAIT cells were shown to significantly increase the proliferation of LM3 and Huh7 HCC cells in vitro. Transcriptomic analyses confirmed that IL-17A induces a PPARα-driven lipid storage program in tumor cells, increasing the intracellular lipid content, as measured by BODIPY staining. As lipid accumulation has been established as a driver of tumor cell proliferation and simultaneously drives MAIT cell dysfunction in metabolic dysfunction-associated steatotic liver disease (MASLD) [11], a bidirectional metabolic mechanism linking immune cytokine production to tumor growth has been identified. The authors corroborate the significance of these findings by correlating a T_H_17-related gene signature with *MKI67* (indicating tumor cell proliferation) and with PPARα/lipid gene expression. In HCC patient cohorts, patients with high T_H_17 or PPARα/lipid signature expression exhibit significantly worse overall survival. In a key experiment, the authors demonstrated that in HCC xenograft models, intratumoral injection of human CD4^+^ MAITs into LM3- or Huh7-bearing NSG mice accelerated tumor growth, which could be reversed by anti-IL-17A antibody blockade.

## Metabolic crosstalk: kynurenine, AHR, glycolysis, and posttranscriptional control of IL-17A

To identify potential molecular mechanisms driving MAIT cell plasticity in HCC, the authors compare transcriptomic data between CD8^+^ and CD4^+^ MAITs. Higher levels of AHR were detected on CD4^+^ MAIT cells than on other subsets, and this level is further elevated in HCC tumors. Stimulation with AHR agonists (FICZ or Kyn) increases glycolytic enzyme expression, glucose uptake, and IL-17A production. Seahorse metabolic flux analysis confirmed higher basal glycolysis and glycolytic capacity in CD4^+^ MAITs than in CD8^+^ or DN MAITs. At the molecular level, glycolysis promotes IL-17A production not by increasing the expression of the *IL17A* mRNA but through posttranscriptional regulation. Together, these findings describe a bidirectional metabolic feedback circuit between liver cancer cells and hepatic/tumor-infiltrating MAIT cells: tumor-derived Kyn → AHR activation in CD4^+^ MAITs → upregulation of glycolysis → IL-17A translation → PPARα/lipid-driven tumor proliferation.

The data by Fu et al. further support the notion that MAIT cells in HCC are neither simply protumor nor antitumor but are context-dependent effectors whose functional fate is shaped by the integration of multiple factors, such as TCR ligand engagement, the cytokine milieu, tumor-derived signals, the metabolic environment, and cellular interactions within the TME. In addition to the previous description of immunosuppressive MAIT_regs_ generated from conventional MAITs [10], the current data indicate that MAIT plasticity generates multiple subsets with tumor-promoting potential through distinct mechanisms. This study has important therapeutic implications: targeting immunosuppressive Kyn, e.g., through IDO1 inhibition, could decrease plasticity toward CD4^+^ MAIT-mediated tumor promotion, whereas anti-IL-17A strategies target downstream effector functions of CD4^+^ MAITs. Whether MAIT cell plasticity can also be reversed, e.g., by combining the use of MAIT TCR ligands and adjuvant treatment that promotes antitumoral T_H_1 responses (such as those involving IL-12 or IL-18), remains to be demonstrated. Overall, the results of the current study convincingly show that targeting the CD4^+^ MAIT–tumor metabolic circuit may represent a new method for improving the effectiveness of HCC immunotherapy.
